# Influence of a High-Impact Multidimensional Rehabilitation Program on the Gut Microbiota of Patients with Multiple Sclerosis

**DOI:** 10.3390/ijms22137173

**Published:** 2021-07-02

**Authors:** Monica Barone, Laura Mendozzi, Federica D’Amico, Marina Saresella, Simone Rampelli, Federica Piancone, Francesca La Rosa, Ivana Marventano, Mario Clerici, Alessia d’Arma, Luigi Pugnetti, Valentina Rossi, Marco Candela, Patrizia Brigidi, Silvia Turroni

**Affiliations:** 1 Department of Medical and Surgical Sciences, University of Bologna, 40138 Bologna, Italy; federica.damico8@unibo.it (F.D.); patrizia.brigidi@unibo.it (P.B.); 2 IRCCS Fondazione Don Carlo Gnocchi, 20148 Milan, Italy; msaresella@dongnocchi.it (M.S.); fpiancone@dongnocchi.it (F.P.); flarosa@dongnocchi.it (F.L.R.); imarventano@dongnocchi.it (I.M.); mario.clerici@unimi.it (M.C.); adarma@dongnocchi.it (A.d.); lpugnetti@dongnocchi.it (L.P.); nutrizione@vrossi.it (V.R.); 3 Department of Pharmacy and Biotechnology, University of Bologna, 40126 Bologna, Italy; simone.rampelli@unibo.it (S.R.); marco.candela@unibo.it (M.C.); silvia.turroni@unibo.it (S.T.); 4 Department of Pathophysiology and Transplantation, Faculty of Medicine and Surgery, University of Milan, 20122 Milan, Italy

**Keywords:** multiple sclerosis, gut microbiota, rehabilitation program, physical activity, immune response, endotoxemia

## Abstract

Multiple sclerosis (MS) is a neurodegenerative inflammatory condition mediated by autoreactive immune processes. Due to its potential to influence host immunity and gut-brain communication, the gut microbiota has been suggested to be involved in the onset and progression of MS. To date, there is no definitive cure for MS, and rehabilitation programs are of the utmost importance, especially in the later stages. However, only a few people generally participate due to poor support, knowledge, and motivation, and no information is available on gut microbiota changes. Herein we evaluated the potential of a brief high-impact multidimensional rehabilitation program (B-HIPE) in a leisure environment to affect the gut microbiota, mitigate MS symptoms and improve quality of life. B-HIPE resulted in modulation of the MS-typical dysbiosis, with reduced levels of pathobionts and the replenishment of beneficial short-chain fatty acid producers. This partial recovery of a eubiotic profile could help counteract the inflammatory tone typically observed in MS, as supported by reduced circulating lipopolysaccharide levels and decreased populations of pro-inflammatory lymphocytes. Improved physical performance and fatigue relief were also found. Our findings pave the way for integrating clinical practice with holistic approaches to mitigate MS symptoms and improve patients’ quality of life.

## 1. Introduction

Multiple Sclerosis (MS) is a chronic disease affecting the central nervous system, characterized by primary demyelination and a variable degree of axonal degeneration mediated by autoreactive immune processes [1]. MS mainly affects individuals in early adulthood and has a dramatic impact on function and quality of life, and no drug can completely prevent or reverse clinical progress. Although the pathogenesis of MS remains unclear, recent studies are providing a better understanding of the genetic, environmental, and lifestyle factors that contribute to the development of this condition [1,2,3,4]. In particular, environmental factors, rather than genetics, have been suggested as key determinants of susceptibility [5]. In this scenario, the human gut microbiota, i.e., the trillion-member microbial community that resides in the gastrointestinal tract, has recently been considered a potential contributing factor to MS etiopathogenesis [6,7,8]. Gut microbes and their bioactive metabolites can affect host immune responses and neurological processes via bidirectional communications with the central nervous system, involving the enteric nervous system and enteroendocrine cells [9,10,11]. In particular, short-chain fatty acids (SCFAs), derived from the gut microbiota fermentation of non-digestible carbohydrates and microbially-produced neurotransmitters, are known to play a crucial role in modulating the endocrine/immune systems and fueling gut-brain exchanges [12,13]. Several works have documented alterations of the gut microbiota in patients with MS (pwMS) [14,15,16,17], identifying the following typical dysbiotic traits: (i) decreased levels of SCFA producers belonging to the Lachnospiraceae family, i.e., *Roseburia*, *Coprococcus*, and *Blautia*); and (ii) increased relative abundances of pathobionts such as *Collinsella*, as well as *Akkermansia*, a mucin degrader involved in pro-inflammatory responses. Such an imbalance has been proposed to result in increased gut and blood-brain barrier permeability, inflammation, and impaired gut-brain connections, which may contribute to the onset and progression of MS [14,18,19,20]. Despite the availability of disease-modifying therapies to reduce the risk of developing relapses [21], there is currently no definitive cure for MS. Especially in the later progressive stages of MS, multidisciplinary rehabilitation programs are prioritized, but access and adherence are still low. To date, none of these programs evaluated the possible variations of the gut microbiota. In an attempt to bridge this gap, we undertook a pilot study on pwMS undergoing a brief high-impact multidimensional rehabilitation program (B-HIPE) in a stimulating leisure environment [22] to evaluate its potential in (i) modulating MS-related dysbiosis and (ii) improving MS symptoms and patients’ quality of life. We enrolled adult pwMS with different disease stages, i.e., diagnosed with both Relapsing-Remitting (RR) and Secondary Progressive (SP) MS, with no changes in disease-modifying drug treatments and no clinical relapse in at least three months prior to enrollment. First, we profiled the gut microbiota of pwMS at baseline to identify disease-related dysbiotic traits by comparison with healthy individuals. Then, we monitored the microbiota trajectories following the B-HIPE program. At the same time, changes in immune responses, intestinal epithelial barrier integrity and clinical parameters, including physical activity and perception of fatigue, were also determined.

## 2. Results

### 2.1. Study Cohort Description

We collected data from 14 pwMS, consisting of 7 males and 7 females. Among these, 10 had a RR MS diagnosis, and 4 had a SP MS diagnosis. Principal descriptive demographic characteristics are shown in Table 1.

### 2.2. B-HIPE Clinical Results: Physical Activity and Diet Adherence

Table 2 reports the clinical results of the B-HIPE program. In particular, a statistically significant difference was observed in the total score of the Modified Fatigue Impact Scale (MFIS-5) (*p* = 0.009, Wilcoxon test), with a better performance after the B-HIPE program. Furthermore, a statistically significant difference was found in the six-Minute Walking Test (6MWT), as well as in the dynamic index calculated through the actigraphic monitoring, with higher values post-intervention (*p* ≤ 0.046). Better adherence to a proper anti-inflammatory diet (see Section 4) was also observed through FFQs, with a statistically significant difference in the total score at the end of the B-HIPE program (*p* = 0.001).

### 2.3. B-HIPE Impact on Immune and Inflammatory Responses: T Lymphocyte Functional Subpopulations, Microbial Translocation, and Gut Permeability

The different T cell subsets were analyzed in pwMS at baseline (T0) and after (T1) the B-HIPE program. The results, as shown in Table 3, indicated that CD4+/IFN-γ+ TH1 and CD4+/ROR-γ+ and CD4+/IL-17+ TH17 were significantly decreased in pwMS after the rehabilitation program. No significant differences were observed in the other immunological parameters analyzed.

We also found a significant decrease in the serum concentration of lipopolysaccharide (LPS) after B-HIPE (median: T0 = 0.8 EU/mL, T1 = 0.6 EU/mL; *p* = 0.02). Conversely, the intestinal fatty acid-binding protein (I-FABP) was significantly increased at T1 (median: 1114 pg/mL) compared to T0 (median: 628 pg/mL; *p* = 0.002). LPS translocates from the intestinal lumen to the peripheral circulation when the integrity of the gastrointestinal barrier is altered, while I-FABP is released into circulation in case of enterocyte damage and intestinal ischemia. The detection of serum cortisol, a neuroendocrine indicator of the hypothalamic-pituitary-adrenal axis activity, involved in the integration of the body’s stress response with immune activity [23,24], showed no statistical difference after the rehabilitation program. These data are shown in Figure S1.

### 2.4. B-HIPE Impact on the Gut Microbiota

#### 2.4.1. Gut Microbiota Dysbiosis at Baseline

**Figure 1:**
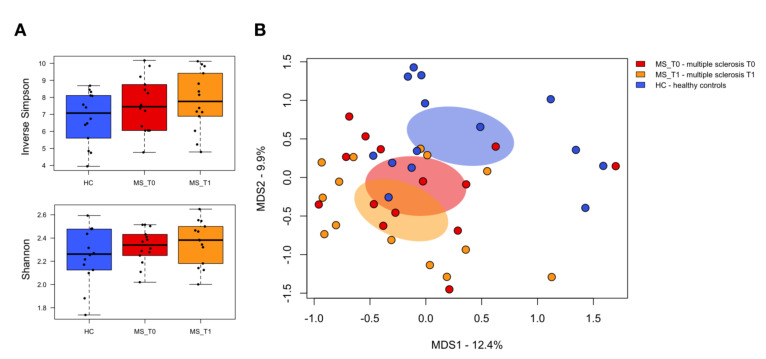
The gut microbiota diversity of multiple sclerosis patients before and after the rehabilitation program compared to healthy subjects. (**A**) Boxplots showing the distribution of alpha diversity, measured using the Inverse Simpson (top) and Shannon (bottom) indices, for the gut microbiota of multiple sclerosis patients before (MS_T0, red) and after the rehabilitation program (MS_T1, orange), as well as for age- and sex-matched healthy subjects across Italy (HC, blue). (**B**) Principal Coordinates Analysis (PCoA) of the gut microbial communities, based on the Jaccard similarity index. Significant segregation between study groups was found (*p* < 1 × 10^−4^, permutation test with pseudo-F ratios).

The bacterial 16S rRNA gene-based next-generation sequencing yielded a total of 1,215,494 high-quality reads, with an average of 43,410 ± 10,522 sequences per sample, binned into 3238 ASVs. No significant differences were found in alpha diversity measures between pwMS at baseline (MS_T0) and age-/sex-matched healthy individuals from the same geographical location, i.e., across Italy (HC) (*p* ≥ 0.3; Wilcoxon test) (Figure 1A). No differences were observed even when stratifying MS patients by clinical course (i.e., RRMS vs. SPMS) (Figure S2). In contrast, Principal Coordinates Analysis (PCoA) of inter-individual variation, based on Jaccard similarity, revealed significant segregation between MS_T0 and HC (*p* ≤ 1 × 10^−4^; permutation test with pseudo-F ratio) (Figure 1B), but again, no significant differences were observed when grouping MS patients according to the clinical course (*p* = 0.08) (Figure S2). Similarly, no separation was found in the whole cohort by major microbiota-associated confounding factors, such as age and gender (Figure S3).

**Figure 2:**
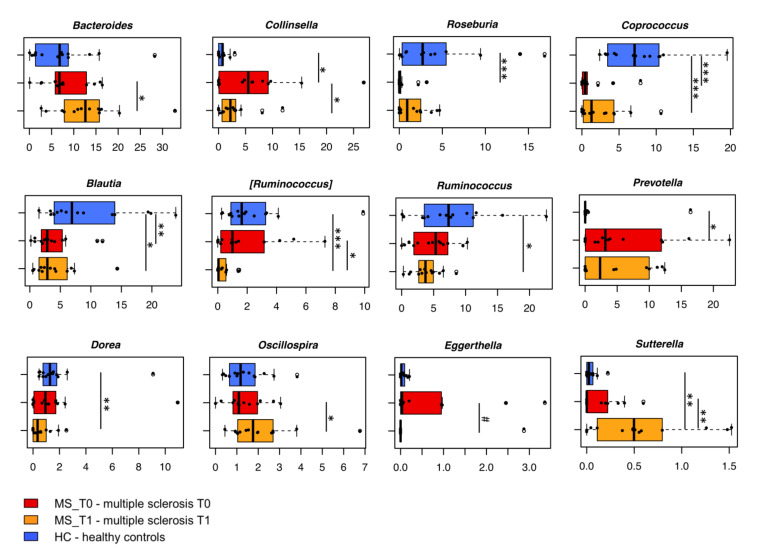
The dysbiotic layout of the gut microbiota in multiple sclerosis patients is partially recovered following the rehabilitation program. Boxplots showing the relative abundance distribution of bacterial genera significantly different between study groups (multiple sclerosis patients before (MS_T0, red) and after the rehabilitation program (MS_T1, orange), and age/sex-matched healthy subjects across Italy (HC, blue)). For *Eggerthella*, only a trend was observed. *, *p* ≤ 0.05; **, *p* ≤ 0.01; ***, *p* ≤ 0.001; #, *p* ≤ 0.1; Wilcoxon test.

When focusing on the taxonomic variations in the gut microbiota of MS_T0 compared to HC (Figure 2, Figures S4 and S5), we found significantly reduced levels of Firmicutes in the former (mean relative abundance ± sem in MS_T0 vs. HC, 59.8% ± 3.3% vs. 79.0% ± 3.0%; *p* = 0.005, Wilcoxon test), together with increased proportions of Bacteroidetes (19.1% ± 2.4% vs. 11.0% ± 2.7%; *p* = 0.03), Actinobacteria (14.7% ± 2.4% vs. 7.0% ± 1.5%; *p* = 0.008) and Proteobacteria (4.5% ± 1.9% vs. 0.5% ± 0.3%; *p* = 0.003). At family level, the MS-associated gut microbiota was significantly depleted of the typical dominant families *Lachnospiraceae* (18.1% ± 2.0% vs. 35.1% ± 4.9%; *p* = 0.002) and *Ruminococcaceae* (20.4% ± 2.3% vs. 30.6% ± 4.0%; *p* = 0.05), while enriched in *Coriobacteriaceae* (9.5% ± 1.8% vs. 1.4 ± 0.4%; *p* = 0.0008), *Veillonellaceae* (5.3% ± 1.5% vs. 0.9% ± 0.3%; *p* = 0.02), *Prevotellaceae* (5.9% ± 1.9% vs. 1.2% ± 1.2%; *p* = 0.02) and *Enterobacteriaceae* (4.1% ± 1.9% vs. 0.3% ± 0.2%; *p* = 0.04). In agreement with our previous work and currently available literature [16,25,26], the genus-level gut microbiota layout of MS_T0 showed several dysbiotic signs, including a dramatic depletion of SCFA-producing genera, such as *Roseburia*, *Coprococcus* and *Blautia* (*p* ≤ 0.002), and an increase in *Collinsella* (8.7-fold) and *Prevotella* (4.5-fold) (*p* ≤ 0.02), compared to HC (Figure 2). Both *Collinsella* and *Prevotella* have previously been associated with autoimmune disorders and increased levels of IL-17A [27,28].

#### 2.4.2. B-HIPE-Related Modulation of the Gut Microbiota

With specific regard to the B-HIPE impact, no differences were observed in terms of intra- and inter-sample variability (*p* ≥ 0.3; Wilcoxon test and permutation test with pseudo-F ratios, respectively) (Figure 1), but a major rearrangement in the gut microbiota composition. In particular, the compositional changes were already appreciable at the phylum level, with a significantly reduced relative abundance of Actinobacteria (MS_T0 vs. MS_T1, 14.7% ± 2.4% vs. 9.3% ± 2.4%; *p* = 0.02, Wilcoxon test), whose post-rehabilitation values approached those observed in HC (Figures S4 and S5). On the other hand, MS_T1 still maintained lower levels of Firmicutes and higher levels of Bacteroidetes and Proteobacteria compared to HC (*p* ≤ 0.002), suggesting an overall greater resilience of members of these phyla to be affected by the rehabilitation program. At the family level, MS_T1 showed a significant reduction in Coriobacteriaceae and Peptostreptococcaceae (*p* ≤ 0.02), as well as an enrichment of Bacteroidaceae and [Barnesiellaceae] (*p* ≤ 0.05). However, lower levels of Lachnospiraceae (*p* ≤ 0.05) and higher levels of Veillonellaceae, Enterobacteriaceae, and Rikenellaceae (*p* ≤ 0.04) still persisted. Interestingly, the genus-level (Figure 2) MS_T1 gut microbiota showed a 2.2- and 6-fold depletion of *Collinsella* and [*Ruminococcus*], together with an enrichment of *Bacteroides*, *Sutterella*, and *Oscillospira* (*p* ≤ 0.04). A decreasing trend in *Eggerthella* was also observed (*p* = 0.08). Despite no statistical significance (*p* = 0.2), *Coprococcus* underwent an appreciable 2.1-fold increase compared to MS_T0, but its proportions were still lower than those of HC (*p* = 0.005). On the other hand, *Blautia* levels remained relatively stable over time, while those of other SCFA producers, namely *Ruminococcus* and *Dorea*, underwent a decrease, becoming significantly lower than HC (*p* = 0.04).

#### 2.4.3. Correlations between Gut Microbiota Profiles and Host Covariates

**Figure 3:**
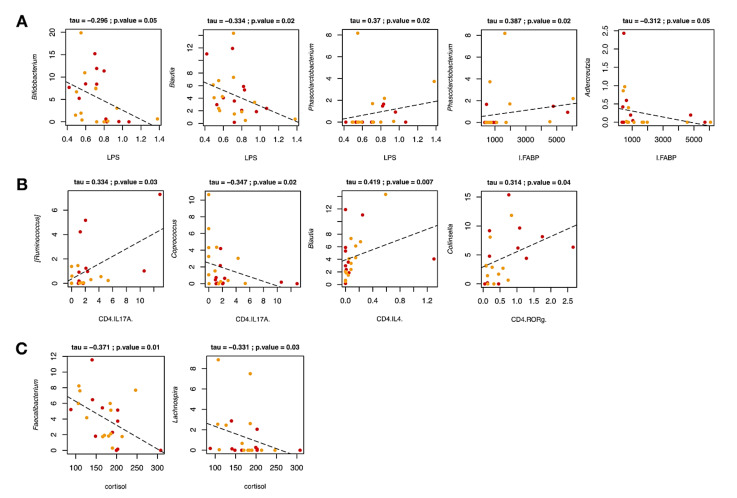
Associations between genus-level relative abundances and levels of LPS and I-FABP (**A**), lymphocytes subpopulations (**B**), and cortisol (**C**) in multiple sclerosis patients over the rehabilitation program. Only statistically significant correlations (*p* ≤ 0.05) with an absolute Kendall rank correlation coefficient ≥0.3 for genera with relative abundance ≥1% are shown.

Correlations between the relative abundances of bacterial taxa and lymphocyte subpopulations, as well as serum levels of LPS and I-FABP in pwMS across B-HIPE were next specifically sought (Figure 3). Interestingly, LPS was negatively correlated with the well-known probiotic genus, *Bifidobacterium* (*p* = 0.05, tau = −0.296, Kendall rank correlation test), as well as *Blautia* (*p* = 0.02, tau = −0.334), whose probiotic properties have recently been debated [29]. On the contrary, LPS and I-FABP showed a positive correlation with the Gram-negative bacterium *Phascolarctobacterium* (*p* = 0.02, tau = 0.370 and 0.387, respectively). Moreover, I-FABP was negatively correlated with *Adlercreutzia* (*p* = 0.05, tau = −0.312).

When focusing on T lymphocyte subpopulations (Figure 3), we found that CD4+/IL-17A+ TH17 levels were positively correlated with the relative abundance of [*Ruminococcus*] (*p* = 0.03, tau = 0.334), while negatively with that of *Coprococcus* (*p* = 0.02, tau = −0.347). It should be noted that the proportions of the former underwent a depletion after rehabilitation, while the latter tended to increase. As for the lymphocyte subpopulation CD4+/IL-4+ TH2, a positive correlation with *Blautia* was observed (*p* = 0.007, tau = 0.419). Finally, a positive correlation between CD4+/ROR-γ+ TH17 levels and *Collinsella* was found (*p* = 0.04, tau = 0.314), suggesting that the potential detrimental role played by this bacterial genus could be linked to enhanced ROR-γ expression in TH17 lymphocytes, ultimately supporting autoimmune neuroinflammation [30,31,32].

Correlations between genus-level relative abundances and serum levels of relevant metabolites, including SCFAs and cortisol, were further assessed (Figure 3). Interestingly, a negative correlation was found between cortisol and the health-promoting SCFA producers *Faecalibacterium* and *Lachnospira* (*p* = 0.01 and 0.03, tau = −0.371 and −0.331, respectively). It should be noted that cortisol levels did not undergo significant variations across the duration of the study, while the amounts of some SCFA producers increased or tended to increase. It is therefore tempting to speculate that a longer program duration may lead to a decrease in cortisol levels.

## 3. Discussion

Herein we dissected the influence of a brief high-impact multidimensional program on the gut microbiota of pwMS, with a medium-severe level of disability (following the EDSS score) and a long disease course, evaluating both the extent of the initial dysbiosis and the dynamic changes induced by the B-HIPE program. As commonly observed in the literature [16,33,34], the gut microbiota of pwMS at enrollment (i.e., before entering B-HIPE) displayed some dysbiotic features compared to healthy age/gender-matched individuals. In particular, pwMS were dramatically depleted of typically health-associated SCFA-producing bacteria belonging to the *Lachnospiraceae* family, i.e., *Roseburia*, *Coprococcus*, and *Blautia*. This is a feature common to many disorders, probably resulting from disruptions in the redox state of the intestine [35,36]. In the context of MS, a decreased production of SCFAs, and especially butyrate, could be closely associated with disease-related inflammation, given the crucial involvement of this microbial metabolite in driving Treg differentiation [37,38] and in maintaining the integrity of the intestinal epithelial barrier [39,40,41]. On the other hand, the microbial ecosystem of pwMS at enrollment was particularly enriched in *Collinsella* and *Prevotella*, both associated with autoimmune disorders and increased levels of the pro-inflammatory cytokine IL-17A [27,28]. The overabundance of *Collinsella* has already been observed in pwMS and is supposed to exacerbate symptoms by stimulating pro-inflammatory responses and compromising barrier integrity, thus aggravating a state of chronic inflammation [16,42,43]. The increased presence of *Prevotella* spp. has also been associated with inflammatory disorders, such as rheumatoid arthritis and metabolic diseases [25,44,45,46], where it could play a role in sustaining inflammatory tone through the stimulation of TH17 and CD8+ T cells-derived pro-inflammatory cytokines (i.e., IFN-γ and TNF-α) [47,48,49]. Nevertheless, the role of *Prevotella* in modulating host physiology is still controversial. Recent work indicates that *Prevotella histicola* can suppress experimental autoimmune encephalomyelitis (EAE) in the murine model as the disease-modifying drug Copaxone [8], suggesting its potential as a microbial monotherapy for the treatment of MS [50].

The pwMS enrolled in our study have participated in B-HIPE, a brief high-impact multidimensional rehabilitation program implemented for the promotion of a proper lifestyle in MS. Specifically, B-HIPE focused on the interplay between competence, motivation, and opportunity to increase in a short time awareness and motivation of pwMS. To this end, physiotherapy, mindfulness, sailing, healthy eating, and cultural activities were experienced in a leisure environment of a seaside village at La Maddalena (Sardinia, Italy). Based on clinical results, our B-HIPE program impacted fatigue and walking, with a statistically significant difference pre vs. post-intervention in both variables. The result in terms of fatigue improvement is particularly relevant, considering the strong impact that this disturbance has on the daily life of pwMS. In fact, fatigue is one of the most challenging symptoms of pwMS, and the literature is focusing on the importance of early interventions. Furthermore, it has been considered that the pharmacological treatment of fatigue has poor efficacy. In contrast, lifestyle and specifically physical activity may have some clinical benefits [51,52]. In this regard, with our brief high-impact program, we obtained a statistically significant improvement also in terms of distance covered in the 6MWT, which has a considerable impact on the daily life of pwMS as well. These findings could be linked with the contents of the B-HIPE program: in fact, it is well known that an anti-inflammatory diet plays a key role in the management of the disease [53,54]. The relationship between nutrition and fatigue is also described in the literature [55] but changing dietary habits is a complex challenge to face [56]. Our FFQ-based results demonstrated that pwMS, after participating in our program, showed very high adherence to the proposed anti-inflammatory diet in contrast to the baseline. This result is particularly significant considering the impact of an anti-inflammatory diet on MS on both immunological parameters [26] and the gut microbiota [16].

As for the latter, when monitoring the dynamics in pwMS across B-HIPE, we detected no significant alterations in intra- and inter-individual variability, suggesting a limited impact on the overall microbiota structure. However, a compositional rearrangement was found, indicating a partial recovery of the initial dysbiosis. In particular, the rehabilitation program resulted in reduced proportions of bacteria belonging to the Actinobacteria phylum and Coriobacteriaceae family, namely *Collinsella*, well-known for its overrepresentation in MS-related microbiota layouts and its association with immune markers such as TH17 [57,58], as discussed above. It should be noted that by the end of the program, Actinobacteria levels approached those observed in healthy individuals. At the genus level, we also observed the depletion of *Ruminococcus* (pro-inflammatory mucolytic taxon from the *Lachnospiraceae* family), along with increased amounts of health-associated SCFA producers, such as *Coprococcus*, *Bacteroides*, and *Oscillospira*, suggesting a partial restoration of an anti-inflammatory SCFA-producing microbiota layout. In addition, we found a decreasing trend for *Eggerthella*, a bacterial genus that has been identified as a potential biomarker of patients with autoimmune disorders, such as MS and rheumatoid arthritis [28,42,59].

Consistent with the hypothesis of a reduced inflammatory tone following B-HIPE, CD4+/IFN-γ+ TH1 and CD4+/ROR-γ+ and CD4+/IL-17+ TH17 underwent a significant decrease, as did circulating LPS. This suggests integrity of the intestinal barrier and, therefore, a reduced translocation of Gram-negative bacteria into the bloodstream. On the other hand, we found an increase in serum levels of I-FABP, a potential biomarker of tissue damage following enterocyte necrosis [60]. However, levels of this protein may not necessarily reflect enterocyte loss nor be directly correlated with inflammatory status, as recently discussed [61]. As expected, a negative correlation was observed between serum LPS levels and the proportions of the health-promoting genera *Bifidobacterium* and *Blautia*. Several probiotic strains, including *Bifidobacterium* spp., have, in fact, been associated with reduced endotoxemia through improved barrier function [62,63]. When focusing on T lymphocyte subpopulations, CD4+/IL-17+ TH17 levels were negatively correlated with the relative abundance of *Coprococcus*, already reported as depleted in MS patients [16,64], and which tended to increase at the end of the B-HIPE program. Conversely, the levels of the aforementioned lymphocyte subpopulation were positively correlated with [*Ruminococcus*]—whose relative abundance decreased following B-HIPE—supporting its involvement in the typical inflammatory tone found in MS patients and EAE murine models [65]. Interestingly, the relative abundance of *Collinsella* was positively correlated with CD4+/ROR-γ+ TH17 levels, suggesting the ability of this bacterial genus to support autoimmune neuroinflammation by inducing IL-17 production by ROR-γ+ TH17 cells [30,66]. The proposed mechanism through which TH17 and IL-17 modulate neuroinflammation involves the expression of a functional IL-17 receptor A on astrocytes [67] and the upregulation of inflammatory cytokine and chemokine production [68]. In murine EAE models, impaired IL-17-mediated signaling in astrocytes has been found to ameliorate EAE [69]. Moreover, TH17 and IL-17 inhibit both maturation and survival of oligodendrocytes [70], as well as their apoptosis [71]. In MS, neurodegeneration linked to persistent demyelination is often associated with impaired apoptosis of oligodendrocytes caused by direct cytotoxicity from both antigen-specific T cells and the activation of resident microglia [72]. It is therefore tempting to speculate that the B-HIPE-induced decline in some pathobionts, such as *Collinsella* and [*Ruminococcus*], along with the greater potential to produce SCFAs, could help alleviate MS-related inflammation, with systems-level benefits.

Despite the small sample size, the data obtained in our pilot study underline the importance of carefully monitoring the most relevant clinical and psychological aspects in pwMS, focusing on physical activity, nutrition, immune and inflammatory response, and the gut microbiome. MS profoundly reduces the quality of life, hindering the ability to work and carry out social activities. Although treatments currently in use are unable to cure the disease or reverse its progression, our multidimensional approach has proven to be effective in balancing some MS symptoms and contributing to significantly improving patients’ quality of life, even in such a short period of time. This is not surprising given the intrinsic characteristics of B-HIPE, high motivation and impact, and the modifiable nature of the gut microbiota, capable of being influenced even by short-term interventions [73,74]. While it is necessary to validate these promising findings in larger cohorts, possibly homogenous for variables of clinical relevance, especially disease course, integrating such an approach into typical clinical practice could address important needs of pwMS, supporting the delivery of high-quality care to improve patient outcomes and potentially reduce direct and indirect costs at the same time.

## 4. Materials and Methods

### 4.1. Subject Enrollment and Rehabilitation Program

A total of 14 pwMS were consecutively recruited from the Multiple Sclerosis Center—Neuromotor Rehabilitation Unit of Don Carlo Gnocchi Foundation, IRCCS in Milan (Italy). Inclusion criteria were: (1) diagnosis of RR and SP MS; (2) age ≥ 18 and ≤70 years; (3) no change of pharmacological disease-modifying treatments in the six months prior to enrollment; (4) no clinical relapse or use of steroid treatment in the three months prior to enrollment; (5) pwMS who were on a Western diet; (6) pwMS with motor control of upper limbs sufficient to maneuver a tiller; (7) provided informed consent for study participation. Exclusion criteria included: (1) history of nervous system disorders other than MS; (2) unstable psychiatric illness, such as psychosis or major depression; (3) severe disability with EDSS score > 8; (4) severe cognitive impairment (i.e., dementia), according to the patient’s medical records; (5) severe visual impairment; (6) alcohol and drugs abuse; (7) dysphagia and/or comorbidities requiring protected environments and specific medical assistance. The study was conducted in compliance with the Helsinki Declaration of 1975, as revised in 2008. Local Ethics Committee (Don Carlo Gnocchi Foundation) approved the study (number 07_23/05/2018), and written informed consent to be included in the study was obtained from participants before study initiation. Details of the B-HIPE rehabilitation program were described in our previous paper [22]. In brief, B-HIPE is a one-week program in which several activities are integrated:

(a) Neuromotor rehabilitation in individual and group sessions adapted to each participant’s specific needs and functional limitations;
(b) Recommended diet mainly based on the Mediterranean diet principles, including fresh fruits and vegetables, whole grain products, legumes, nuts and seeds, fish, eggs, and a small amount of poultry and dairy products. On the contrary, red meat, processed meat, alcoholic and sweet drinks were excluded;
(c) Sailing course proposed with equipped single- and double-seated monohulls designed to accommodate disabled sailors;
(d) Mindfulness, through group sessions taking place in the late afternoon hours on a quiet beach, with participation extended to all staff members.

### 4.2. Assessment of Clinical and Nutritional Variables

At baseline (T0) and after intervention (T1), clinical and nutritional tests/questionnaires were administered to all study participants:

(a) An exercise test used to assess aerobic capacity and endurance (six-Minute Walking Test, 6MWT) [75]; the body sway and dynamics of walking were recorded by an instrumental assessment with actigraphs;
(b) A questionnaire to measure fatigue (Modified Fatigue Impact Scale, 5-item version, MFIS-5) [76];
(c) A Food Frequency Questionnaire (FFQ), administered during a face-to-face interview with a team of professional nutritionists to evaluate the adherence to the diet.

The FFQ used is an adapted version of the validated 14-point Mediterranean Diet Adherence Screener [77]. The FFQ consisted of 19 questions on the frequency of food consumption (fruit, vegetables, olive oil, red meat, processed meat, fish, legumes, alcoholic drinks, commercial sweet, nuts, and dairy). Each question included three possible answers and scores ranging from 0 to 2. The FFQ final score ranged from 0 to 38, where 38 represented the highest adherence to our recommended diet.

### 4.3. Serum/Blood Analysis

#### 4.3.1. Blood Sample Collection and Cell Separation

At enrolment and after the rehabilitation program, whole blood (10 mL) was collected in vacutainer tubes containing ethylenediaminetetraacetic acid (EDTA) (Becton Dickinson & Co., Rutherford, NJ, USA). Peripheral blood mononuclear cells (PBMCs) were separated using the lympholyte separation medium (Cedarlane, Hornby, Ontario, CA, USA) and washed twice in PBS at 1500 rpm for 10 min; viable leukocytes were determined using a Scepter 2.0 Handheld Automated Cell Counter (Millipore, Billerica, MA, USA).

#### 4.3.2. Serum

Serum was collected in vacutainer tubes containing serum separator (Becton Dickinson & Co., Rutherford, NJ, USA), centrifuged at 3000 rpm for 10 min, and stored at −80 °C until use.

#### 4.3.3. Intracellular Cytokine or Transcription Factor Staining in PBMCs

Lymphocyte subsets were analyzed in freshly isolated PBMCs that were incubated for 30 min at 4 °C in the dark with Phycoerythrin-Cyanin-5 (PC5)-labeled anti-CD4 (clone SFCI12T4D11, mouse IgG1; Beckman-Coulter, Brea, CA, USA), PC7-labeled, Phycoerythrin-Texas Red (ECD)-labeled anti-CD25 (clone B1.49.9, mouse IgG2a; Beckman-Coulter). After incubation, the cells were washed, treated with the Cell Permeabilization kit (FIX & PERM kit; eBioscience, San Diego, CA, USA), and incubated for 30 min at 4 °C in the dark with the following PE-labeled monoclonal antibodies: anti-IL-10 (clone JES9D7, mouse IgG1; R&D Systems, Minneapolis, MN, USA), anti-TGF-β (clone 9016, mouse IgG1; R&D Systems), anti-IFN-γ (clone 25723, mouse IgG2b; R&D Systems), anti-RORC-γ (clone AFKJS-9, rat IgG2a; eBioscience) anti-GATA-3 (cloneTWAY, rat IgG2B; eBioscience), the FITC-labeled anti-IL-17 (clone BL168, mouse IgG1k; Biolegend), or the Alexa Fluor 488-labeled-anti-FoxP3 (clone 1054C, rabbit IgG; R&D Systems), the FITC-labeled anti-IL-4 (clone MP4-25D2, rat IgG1k isotope; eBioscience) and the PE labelled-anti-T-bet (clone 39D, mouse IgG1 isotype; eBioscience).

#### 4.3.4. Flow-Cytometry Analysis

PBMCs were analyzed using a Beckman-Coulter GALLIOS flow cytometer, equipped with a 22 mW Blue Solid State Diode laser operating at 488 nm and with a 25 mW Red Solid State Diode laser operating at 638 nm, and interfaced with Kaluza analysis software. Two hundred thousand cells were acquired and gated on lymphocyte FSC and SSC properties and considering isotype background, and the following subsets were analysed: CD4+/CD25+/Foxp3+ (Treg); CD4+/IFN-γ+ and CD4+/Tbet+ (TH1); CD4+/ROR-γ+ and CD4+/IL-17+ (TH17); CD4+/TGF-β+, CD4+/BDNF+, CD4+/IL-10+, CD4+/IL-4+, and CD4+/GATA3+ (TH2). The flow cytometry compensation was performed using the fluorescence minus one (FMO) control approach. Briefly, all antibody conjugates in the experiment are included except the one that is controlled for. The FMO measures the spread of fluorescence from the other staining parameters into the channel of interest, determining the threshold for positive staining.

#### 4.3.5. Microbial Translocation and Intestinal Barrier Function

LPS was measured in serum with the LAL Chromogenic Endpoint Assay (Hycult biotechnology, Uden, The Netherlands). I-FABP was measured with an ELISA kit (CUSABIO BIOTECH, Newark, DE, USA) according to the manufacturer’s instructions.

#### 4.3.6. Cortisol Detection

Serum concentrations of cortisol were determined by competitive enzyme immunoassay, the Cortisol Parameter Assay Kit (R&D Systems, Inc.), according to the manufacturer’s recommendations. A plate reader (Sunrise, Tecan, Mannedorf, Switzerland) was used for the assay, and optical densities (ODs) were determined at 450/620 nm. All samples were performed in duplicates. Sensitivity: 0.111 ng/mL; assay range: 0.2–10 ng/mL.

### 4.4. Gut Microbiota Analysis: DNA Extraction and Sequencing

Microbial DNA was extracted from stool samples of pwMS, before and after the rehabilitation program, as previously described [78]. The V3–V4 hypervariable region of the bacterial 16S rRNA gene was PCR-amplified using the 341F and 785R primers [79] with Illumina overhang adapter sequences as previously reported [78]. PCR products of about 460 bp were purified using a magnetic bead-based system (Agencourt AMPure XP; Beckman Coulter) and indexed by limited-cycle PCR using Nextera technology. Indexed libraries, further cleaned up as described above, were pooled at equimolar concentrations, denatured, and diluted to 6 pmol/L. Sequencing was performed on an Illumina MiSeq platform using the 2 × 250 bp protocol, according to the manufacturer’s instructions (Illumina, San Diego, CA, USA). Sequence reads were deposited in the National Center for Biotechnology Information Sequence Read Archive (NCBI SRA; BioProject ID PRJNA739641).

### 4.5. Bioinformatics

Illumina paired-end reads were processed using a pipeline combining PANDAseq [80] and QIIME 2 [81]. High-quality reads were retrieved and clustered into Amplicon Sequence Variants (ASVs) through an open-reference strategy performed with DADA2 [82]. Singleton ASVs and chimeras were filtered out. Taxonomy was assigned using the vsearch classifier [83] against the Greengenes database as a reference (release May 2013). 16S rRNA gene sequencing data of pwMS were compared to publicly available data of healthy age/gender-matched Italian subjects from previous studies (8 subjects, MG-RAST ID: 17761 [78]; 3 subjects, MG-RAST ID: 7058 [84]). Genus-level community composition was generated for all combined cohorts. Alpha diversity was measured using the Shannon and inverse Simpson indices (estimating evenness and richness), while beta diversity was computed based on Jaccard similarity and visualized on a Principal Coordinates Analysis (PCoA) plot. Bar plots were built using the R packages made4 [85] and vegan (http://www.cran.r-project.org/package=vegan/, accessed on 30 April 2021).

### 4.6. Statistical Analysis

The analyses of demographic characteristics and clinical variables were performed with SPSS 24.0. and Jamovi statistical software (The Jamovi Project 2020, v1.8.1). For the description of demographic characteristics, mean, standard deviation (SD), and range for continuous variables are shown as appropriate. To test whether and how our B-HIPE program could impact clinical variables, a statistical comparison between pre (T0) and post-rehabilitation programs (T1) was performed. Differences in immunological and neuroendocrine parameters were assessed by Wilcoxon test.

Regarding the microbiome data, differences in alpha diversity and relative taxon abundance between groups were evaluated by Wilcoxon test, paired or unpaired as needed. The significance of the separation between study groups on the PCoA plot was tested by a permutation test with pseudo-F ratio using the function adonis in the R package vegan [86]. Kendall rank correlation test was used to assess associations between genus-level relative abundances and levels of LPS, I-FABP, lymphocytes subpopulations, SCFAs, and cortisol in pwMS. Statistics were performed using R Studio 1.0.44 on R software v3.3.2 (https://www.r-project.org/, accessed on 30 April 2021); *p*-values were corrected for multiple comparisons using the Benjamini–Hochberg method when appropriate. A *p*-value ≤ 0.05 was considered statistically significant, and a *p-*value ≤ 0.1 a trend.

## 5. Conclusions

Our pilot study demonstrates the potential of a brief high-impact multidimensional rehabilitation program to modulate MS-typical dysbiosis by reducing levels of pathobionts while replenishing SCFA-producing beneficial microbes, thereby partially recovering a eubiotic profile. Such rearrangements could counteract the inflammatory tone typically observed in MS, as evidenced by the reduced circulating levels of LPS and the decrease in pro-inflammatory lymphocyte populations. In parallel, pwMS experienced alleviation of fatigue and an improvement in physical performance. Although future studies are needed to further explore these findings, also in relation to the durability of improvements, our data stress the need to integrate clinical practice with multidimensional approaches to mitigate some MS symptoms and improve patients’ quality of life.

## Figures and Tables

**Table 1:** Demographic characteristics of the enrolled pwMS.

Demographic Characteristics	
N	14
Sex (M/F)	7/7
Age (years) (average ± SD (min–max))	49.43 ± 9.08 (36–69)
MS course (RR/SP)	10/4
Disease duration (years) (average ± SD (min–max))	19.25 ± 5.40 (7–28)
EDSS score (average ± SD (min–max))	5.3 ± 1.66 (2–8)

Abbreviations: N: number; M: male; F: female; MS: Multiple Sclerosis; Min: Minimum; Max: Maximum; RR: Relapsing-Remitting; SP: Secondary Progressive; EDSS: Expanded Disability Status Scale.

**Table 2:** Physical activity parameters and adherence to the recommended diet before (T0) and after (T1) the B-HIPE program. Median, interquartile range, and statistical significance are shown. ns: not significant; Wilcoxon test.

Variable	T0	T1	*p*-Value
MFIS-5	45.00 (10–95)	23.60 (0–60)	0.009
FFQ	14.50 (1–22)	33.86 (28–38)	0.001
6MWT-meters	191.07 (15–380)	260.77 (60–460)	0.002
6MWT-bs/s	0.71 (0.30–1.04)	0.74 (0.49–1.04)	ns
6MWT-di	2.04 (0.14–7.56)	3.43 (0.06–11.26)	0.046

Abbreviations: MFIS-5: higher values indicate greater fatigue; scores are expressed as percentiles. FFQ; food frequency questionnaires to assess the adherence to the B-HIPE program. 6MWT-meters: total distance in meters walked after 6 min. 6MWT-bs/s: a measure of walking speed (body sway/s). 6MWT-di: dynamic index; lower values indicate a more “rigid” walking.

**Table 3:** Percentage of CD4+ T lymphocytes in patients with MS diagnosis before (T0) and after (T1) the B-HIPE program. Median, interquartile range, and statistical significance are shown. ns: not significant; Wilcoxon test.

Subpopulation	T0	T1	*p*-Value
CD4+/CD25+/FOXP3+	0.1	0.03	ns
	(0.05–0.17)	(0.05–0.17)	
CD4+/IFN-γ+	0.18	0.04	0.0004
	(0.1–0.5)	(0.0–0.8)	
CD4+/Tbet+	0.07	0.03	ns
	(0.0–0.1)	(0.02–0.1)	
CD4+/IL-17+	2.0	0.5	0.02
	(1.4–2.3)	(0.0–1.7)	
CD4+/ROR-γ+	0.7	0.3	0.01
	(0.2–1.5)	(0.1–0.6)	
CD4+/IL-4+	0.0	0.08	ns
	(0.0–0.03)	(0.01–0.1)	
CD4+/IL-10+	0.2	0.03	ns
	(0.02–0.2)	(0.0–0.2)	
CD4+/GATA3+	0.4	0.8	ns
	(0.2–1.5)	(0.2–1.4)	
CD4+/TGF-β	0.9	0.5	ns
	(0.4–1.3)	(0.3–0.9)	

## Data Availability

Sequence reads were deposited in the National Center for Biotechnology Information Sequence Read Archive (NCBI SRA; BioProject ID xxx).

## Supplementary Materials

The following are available online at https://www.mdpi.com/article/10.3390/ijms22137173/s1.
