# Supplementary material for: Influence of a High-Impact Multidimensional Rehabilitation Program on the Gut Microbiota of Patients with Multiple Sclerosis
Source: Int J Mol Sci. 2021 Jul 2;22(13):7173. doi: 10.3390/ijms22137173 (PMC8268819; doi:10.3390/ijms22137173)
Supplement: Supplementary file 1 [file ijms-22-07173-s001.zip › ijms-1273198-supplementary.pdf]

Supplementary Materials

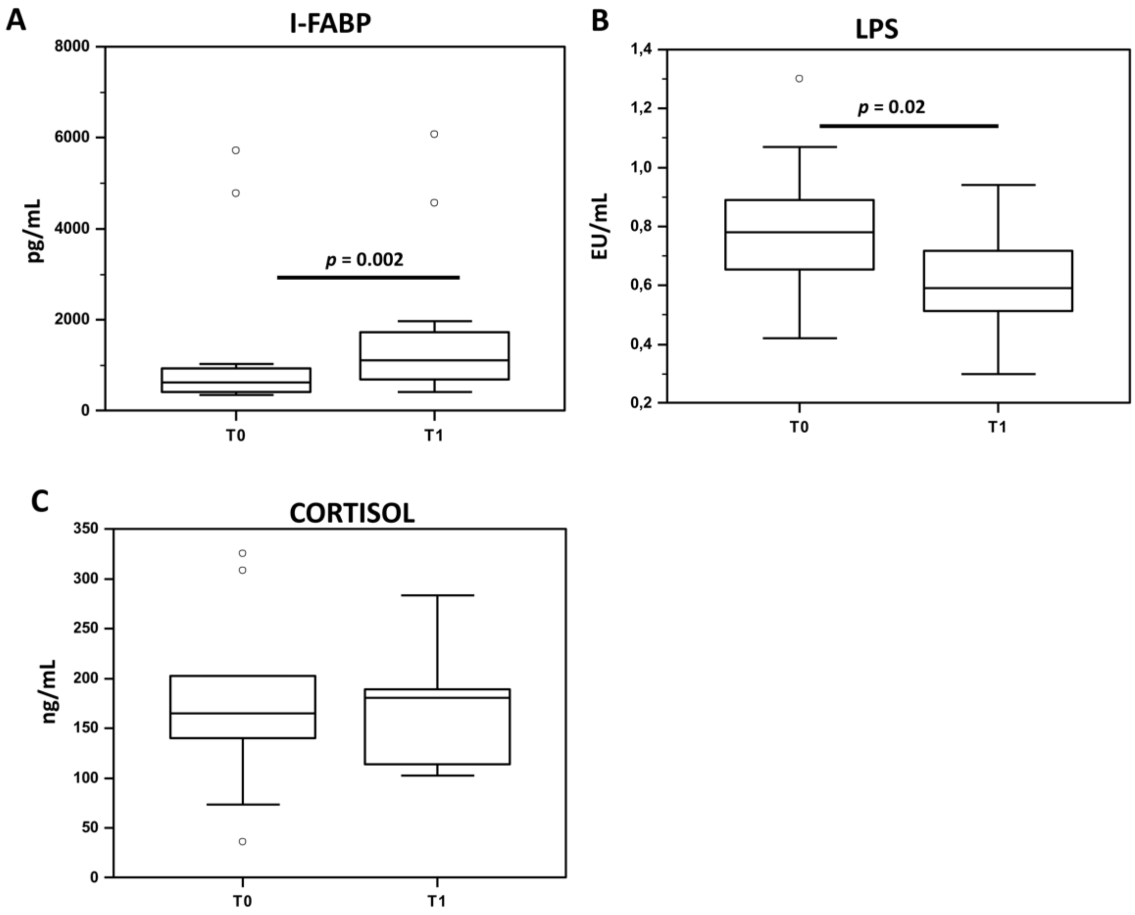

Figure S1. Serum I-FABP, LPS and cortisol concentrations in multiple sclerosis patients before and after a brief high-impact multidimensional rehabilitation program. Boxplots showing the distribution of the amounts of I-FABP (A), LPS (B) and cortisol (C) in the serum of multiple sclerosis patients before (T0) and after (T1) the rehabilitation program. A statistically significant difference was observed for I-FABP and LPS ($p \leq 0.02$, Wilcoxon test).

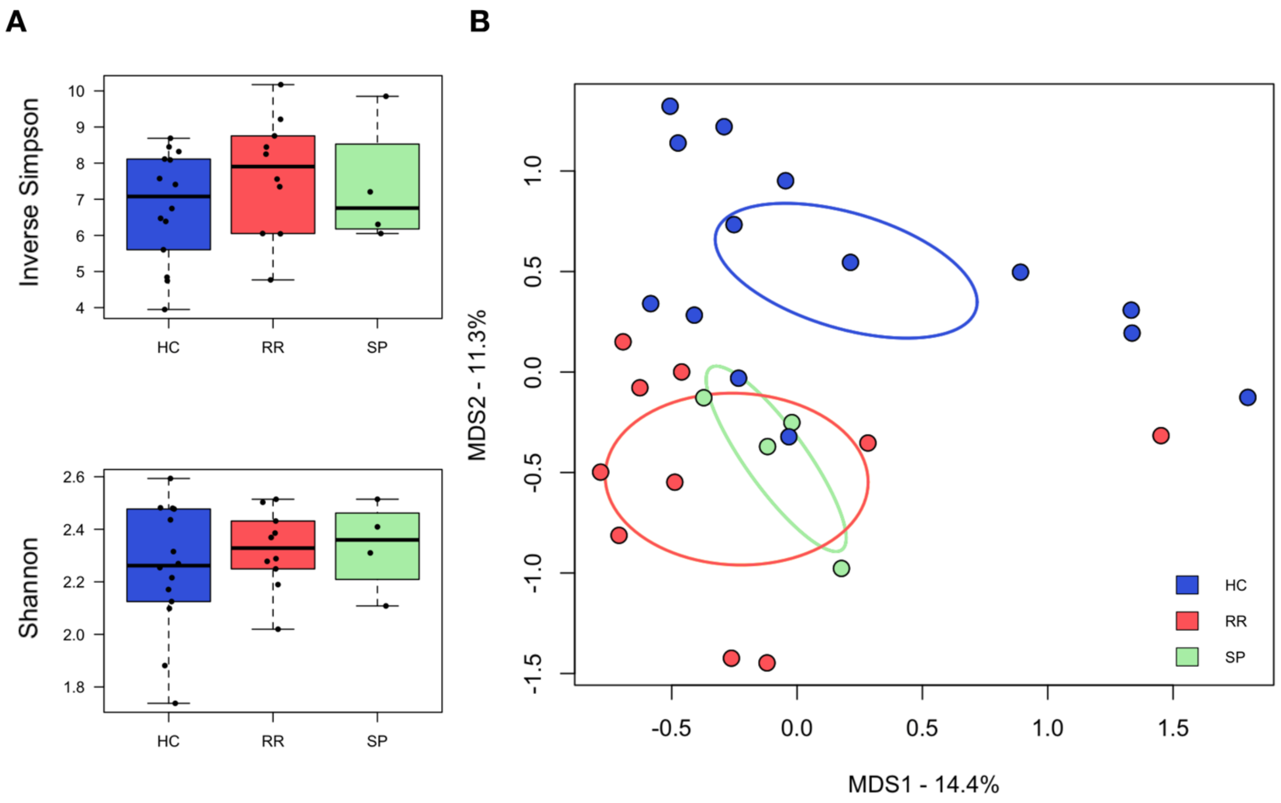

Figure S2. The gut microbiota diversity of relapsing-remitting and secondary-progressive multiple sclerosis patients at baseline compared to healthy subjects. (**A**) Boxplots showing the distribution of alpha diversity, measured using the Inverse Simpson (top) and Shannon (bottom) indices, for the gut microbiota of multiple sclerosis patients with relapsing-remitting and secondary-progressive disease (RR and SP, respectively), as well as for age- and sex-matched healthy subjects across Italy (HC). (**B**) Principal Coordinates Analysis (PCoA) of the gut microbial communities, based on the Jaccard similarity coefficient. A significant separation between multiple sclerosis patients and HC was found regardless of the disease severity ($p \leq 5 \times 10^{-4}$, permutation test with pseudo-F ratios). Only a tendency to segregation was observed between SP and RR patients ($p = 0.08$).

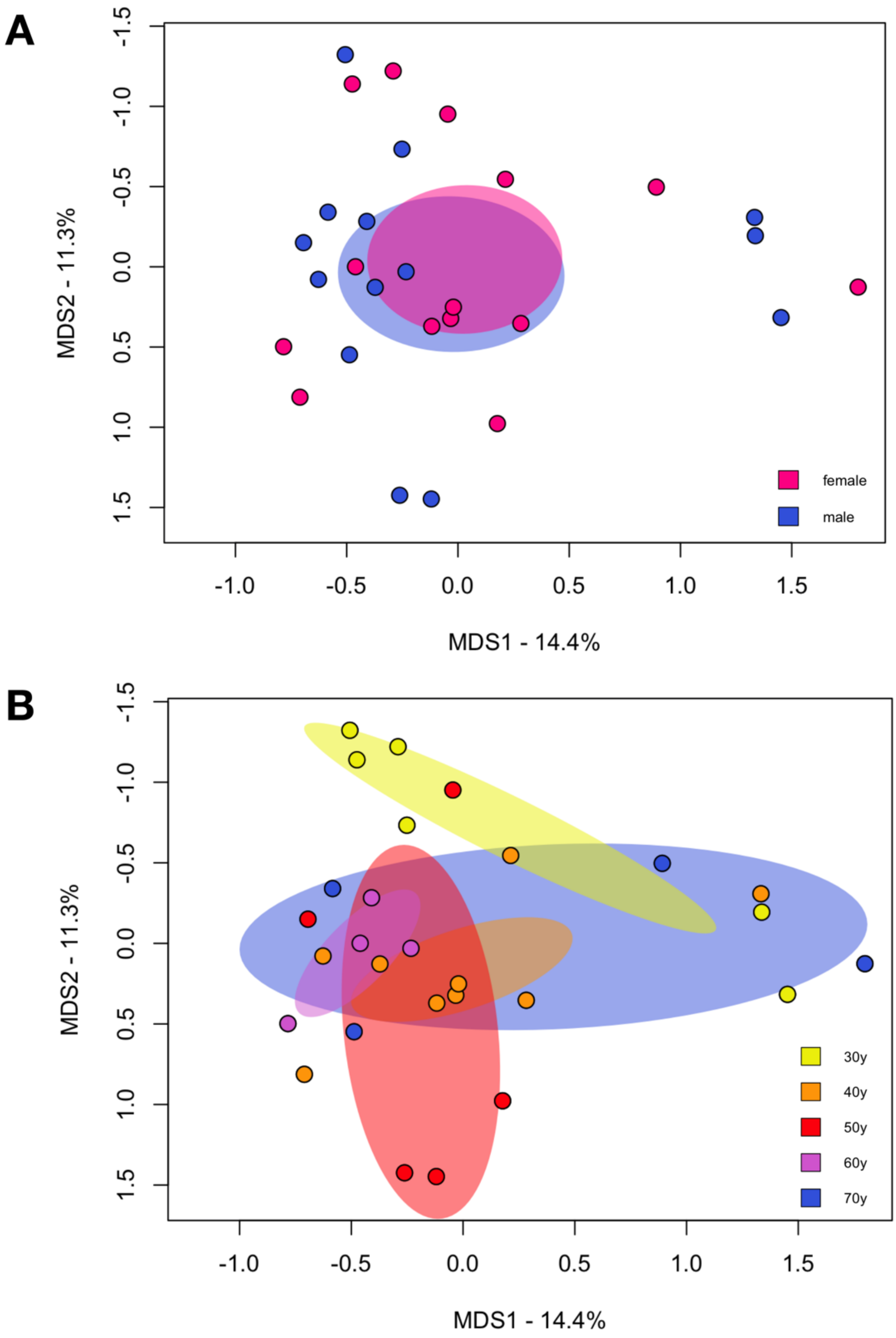

Figure S3. The multiple sclerosis-associated dysbiosis is not affected by microbiota-associated confounding factors. PCoA based on Jaccard similarity coefficient of the gut microbial communities of multiple sclerosis patients and age- and sex-matched healthy subjects across Italy, stratified by gender (female vs male, **A**) and age group (30 to 70 years, **B**). No significant segregation was observed ($p \geq 0.3$, permutation test with pseudo-F ratio). See also Figure 1 and Figure S1.

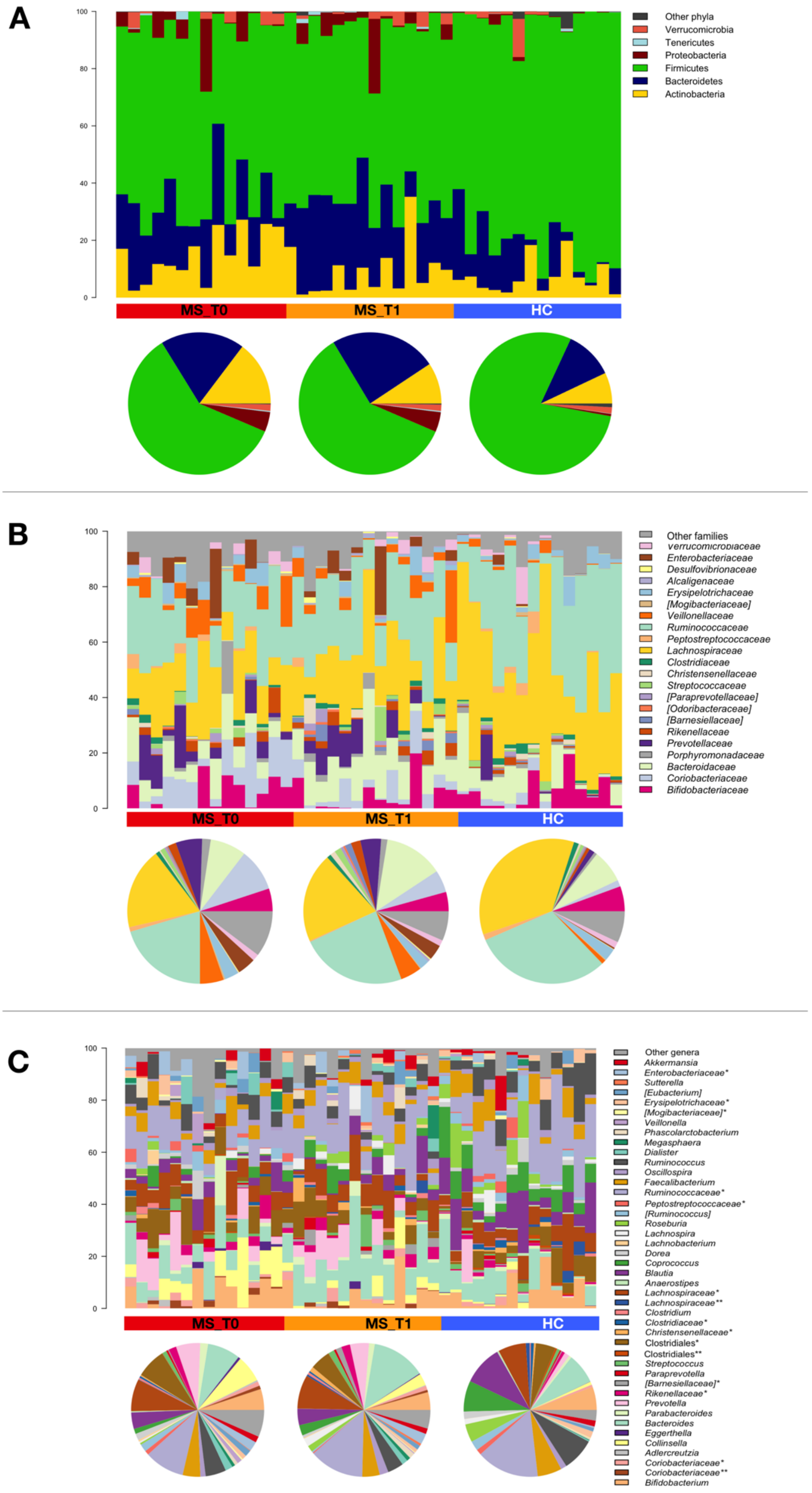

Figure S4. Gut microbiota profiles of multiple sclerosis patients before and after the rehabilitation program, compared to healthy subjects. Bar plots and pie charts summarizing the phylum- (**A**), family- (**B**) and genus-level (**C**) composition of the gut microbiota of multiple sclerosis patients at baseline (MS_T0) and at the end of the rehabilitation program (MS_T1), and of age/sex-matched healthy Italian subjects (HC). Only taxa with relative abundance ≥ 0.5% in at least 10% of samples are represented.

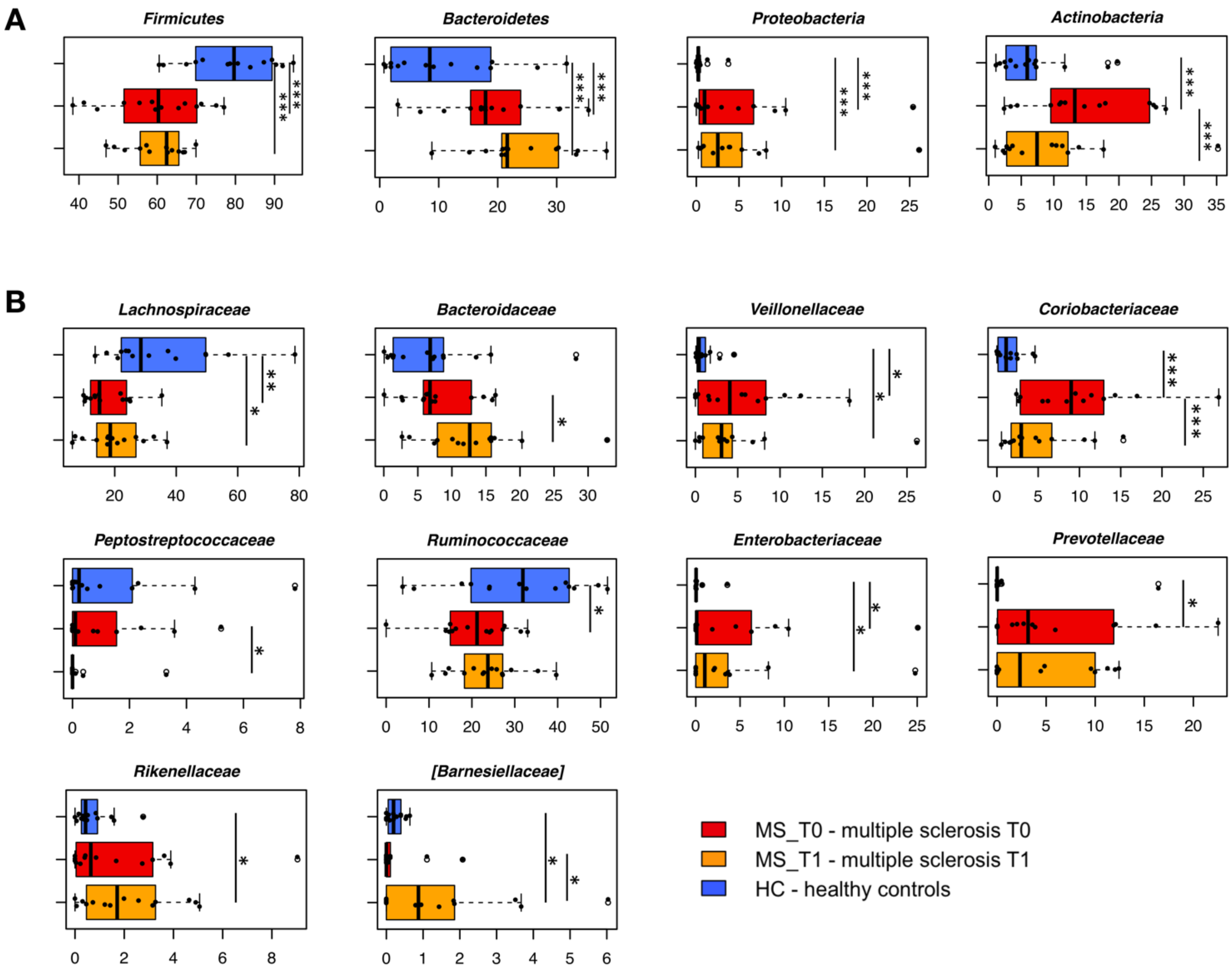

Figure S5. The phylum- and family-level dysbiotic layout in multiple sclerosis patients is partially recovered following the rehabilitation program. Boxplots showing the relative abundance distribution of bacterial phyla (**A**) and families (**B**) significantly different between the study groups [multiple sclerosis patients before (MS_T0, red) and after the rehabilitation program (MS_T1, orange), and age/sex-matched healthy Italian subjects (HC, blue)]. *, $p \leq 0.05$; **, $p \leq 0.01$; ***, $p \leq 0.001$; Wilcoxon test.
